# Melanin formation in barley grain occurs within plastids of pericarp and husk cells

**DOI:** 10.1038/s41598-019-56982-y

**Published:** 2020-01-13

**Authors:** Olesya Yu. Shoeva, Sergey R. Mursalimov, Natalya V. Gracheva, Anastasiya Yu. Glagoleva, Andreas Börner, Elena K. Khlestkina

**Affiliations:** 1 Institute of Cytology and Genetics SB RAS, Novosibirsk, Russia; 2 Volgograd State Technical University, Volgograd, Russia; 3 Leibniz Institute of Plant Genetics and Crop Plant Research, Gatersleben, Germany; 4 N.I.Vavilov All-Russian Research Institute of Plant Genetic Resources, Saint-Petersburg, Russia

**Keywords:** Chloroplasts, Biopolymers

## Abstract

Melanins are a class of darkly pigmented biopolymers which are widely distributed among living organisms. The molecular and cellular mechanisms adopted by bacteria, fungi and animals to synthesize melanin, have been well described, but less is known regarding their production in plants. Here, a pair of barley near isogenic lines, bred to differ with respect to the pigmentation of the spike, was compared in order to understand the tissue and cellular location of melanin deposition. The melanic nature of the pigments purified from black spikes was confirmed by a series of solubility tests and Fourier transform infrared spectroscopy. An analysis of grains harvested at various stages of their development revealed that intracellular pigmented structures first appeared in the pericarp and the husk of black spike plants at early dough stage. The co-localization of these structures with red autofluorescence suggested that they form in chloroplast-derived plastids, here designated “melanoplasts”. Differences in dynamics of plastid internal structure during grain ripening were detected between the lines by transmission electron microscopy. Both lines accumulated plastoglobuli inside plastids, which persisted in black grain pericarp tissue up to the hard dough stage, while neither plastoglobuli nor any plastids were observed in grain of the control line at this stage. The role of plastoglobuli in melanin synthesis is discussed.

## Introduction

Melanin is a dark brown to black pigment present most notably in animal (including human) hair, skin and eyes, but is also found in bacteria, fungi and plants^1–3^. Melanins are synthesized from phenolic precursors, which are oxidized through the action of polyphenol oxidase (PPO) into quinone, which in turn is subsequently polymerized^2,4^. Based on the monomers represented and their mode of synthesis, three classes of melanin are recognized, namely the eumelanins, pheomelanins and allomelanins^2^. Eumelanin, the predominant form found in animals and microorganisms, is produced by oxidative polymerization of tyrosine or phenylalanine into L-3,4-dihydroxyphenylalanine, which is converted into dopachrome and then to melanin^3,5^. The pheomelanins, found only in certain yellow, orange or reddish hair and feathers, are also formed from tyrosine, but contain sulfur^6^. Plant and fungal melanins, classified as allomelanins^2^, are the least well understood, as well as the most heterogeneous group: their precursors are particularly varied^3,7^. In plants, melanins protect against damage from excessive light, but also give mechanical strength to the testa, thereby protecting the developing embryo^8–11^.

In the testa of some *Asparagales* species seed and in the fruits of certain *Compositae* species, melanin is deposited as a layer between hypodermis and the fiber layer^12,13^. At present, the identity of the cellular structures producing the melanin and the cellular processes involved in its secretion and polymerization are unclear. It is even uncertain as to whether the melanin formed in *Compositae* species is chemically similar to what is formed during PPO-induced tissue browning^13,14^. Melanins have been demonstrated as contributing to the dark pigmentation developed by the testa of sunflower, watermelon^1^, tomato^11^, morning glory^15^, oat^7^ and garlic^16^.

The pigment responsible for the black spike formed by certain varieties of barley has long been suspected to be a melanin^17^. The black spike trait is under monogenic control, with the gene responsible (*Blp*) mapping to chromosome 1H^18^. This simple mode of inheritance has facilitated the breeding of lines which are near-isogenic for *Blp*^19^. The objective of the present study was to exploit these near isogenic lines (NILs) to reveal the molecular and cellular basis of melanin formation in barley.

## Results

### Chemical analysis of the pigment in the i:Bw*Blp* grain

**Figure 1:**
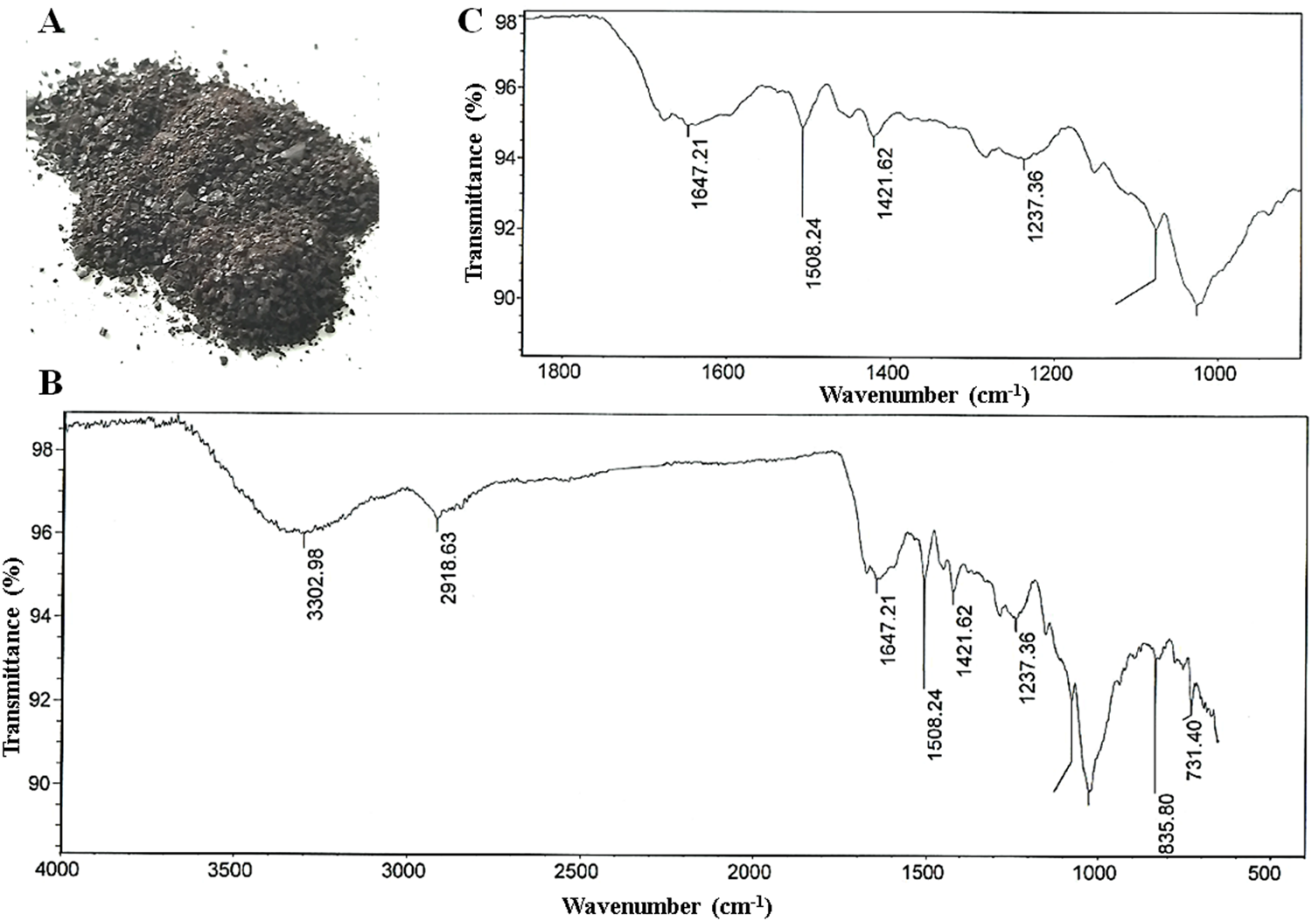
Sample of the black pigment extracted from the husk and pericarp of the i:Bw*Blp* NIL (**A**), FT-IR spectrum of the pigment (**B**) and its expanded fingerprint region (**C**).

The pigment material purified from the i:Bw*Blp* NIL’s grain was a dark, glossy powder (Fig. 1A), which was insoluble in either water or any of the organic solvents, except for hydroxymethylformamide. It was also partially soluble in 76% H_2_SO_4_, and fully soluble in 0.125 M NaOH (Supplementary Table S1). When exposed to either H_2_O_2_ or KMnO_4_, the pigment lost its color, while exposure to FeCl_3_ resulted in the precipitation of a flocculent material which gradually redissolved when the concentration of FeCl_3_ was raised. The behavior of the material was consistent with the presence of quinoid and phenolic compounds. The FT-IR analysis revealed a profile characteristic of melanin (Fig. 1B,C): the broad absorption band in the frequency range 3,200–3,600 cm^−1^ results from the stretching vibrations of the -OH and/or -NH of amide, amine, carboxyl, phenolic or aromatic amino groups in the indole and pyrrole moieties; those in the range 1,200–1,240 cm^−1^ are induced by vibrations of phenol C-O-H groups; those in the ranges 2,850–2,970 and 1,400–1,470 cm^−1^ are related to the stretching and deformation vibrations of aliphatic CH groups; those in the range 1,640–1,650 cm^−1^ are attributed to C = O quinone vibrations, the one at ~1,509 cm^−1^ reflects the stretching vibrations of an aromatic C-C bond; those in the range 1,000–1,075 cm^−1^ are diagnostic of either primary alcohol groups or the С-О-С-bonds present in aromatic ethers.

### Development of the pigmentation during grain filling

By 42 days after sowing, the leading spike of both NILs had emerged fully from the boot, and physiological maturity was reached by 67 days. The black pigmentation first appeared in the grain of the i:Bw*Blp* NIL at the late milk or the early dough stage, beginning at the tip of the spike, then spreading downwards to the base (Supplementary Fig. S1). The pigmentation developed in an uneven manner (Supplementary Fig. S2). It first appeared both as a dark spot in the center of the dorsal side of the grain and as stripes on the palea. By the early dough stage, the pigment began to form under the lemma, by the later dough stage, it increasingly covered the lemmas and paleas at the tip of spike, and finally by the fully ripe stage whole spike become black. Besides the grain, black pigmentation developed in awns first unevenly appeared at the early dough stage and become clearly visible at the hard dough stage (Supplementary Fig. S1).

### The microscopic structure of the developing grains

**Figure 2:**
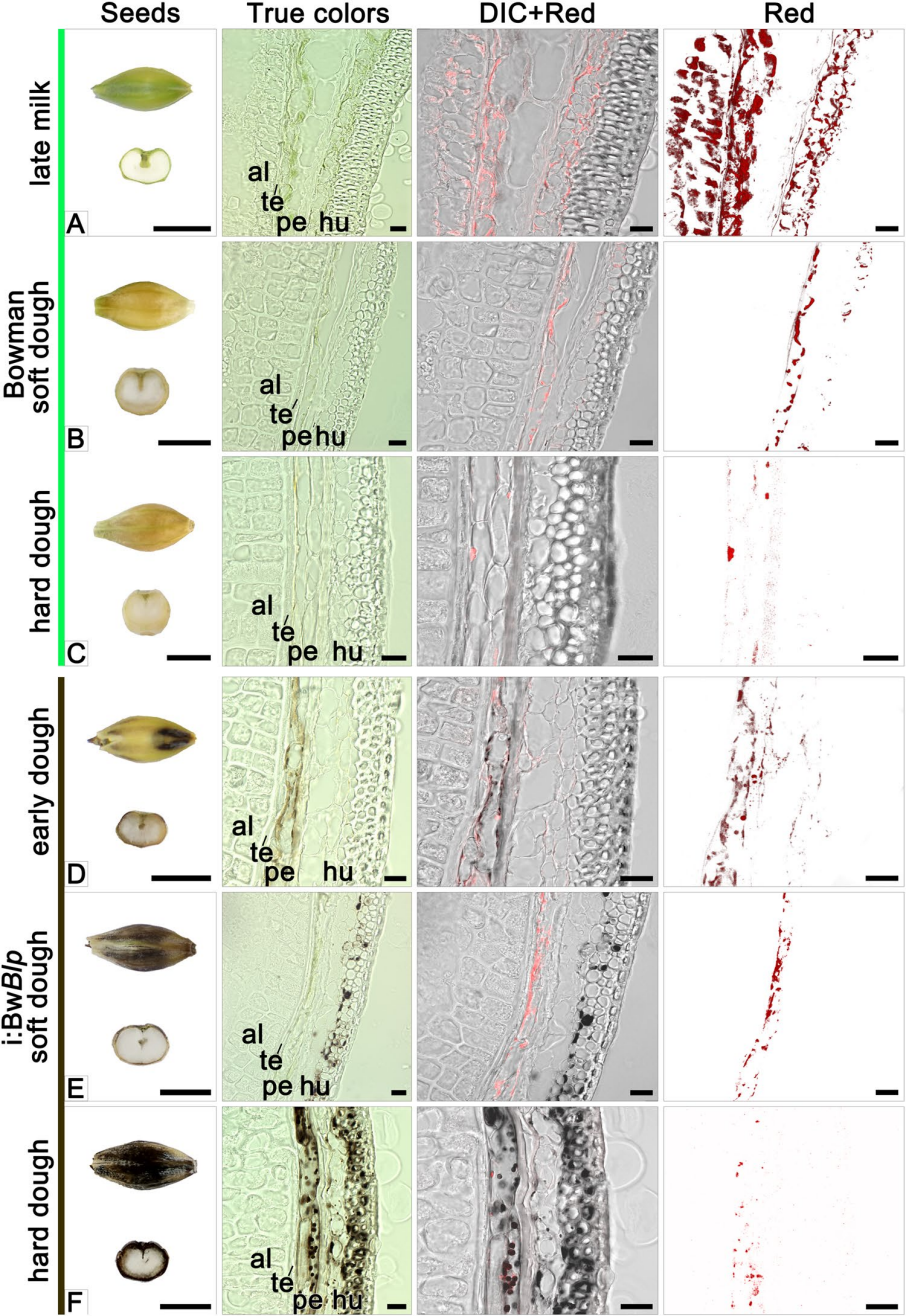
Cross-sections of grain set by cv. Bowman and the i:Bw*Blp* NIL sampled at the late milk, early dough, soft dough and hard dough stages. The images shown illustrate true colors, DIC+ red autofluorescence and red autofluorescence. Scale bar for images of whole grains: 5 mm; and for micrographs: 20 um. Al: aleurone, hu: husk, pe: pericarp; te: testa.

**Figure 3:**
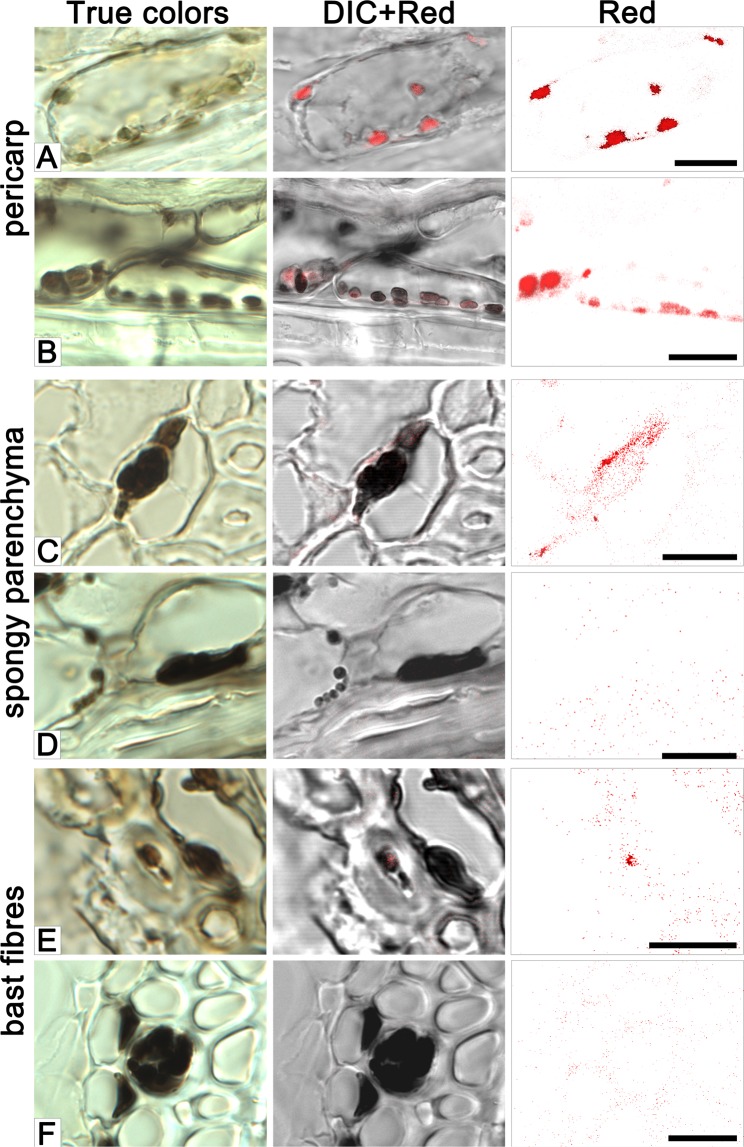
The accumulation of pigment in the pericarp (**A**, **B**), spongy parenchyma (**C**,**D**), and bast fibres (**E**,**F**) cells of the iBw*Blp* NIL’s husk. Samples taken at the early (**A**,**C**,**E**) and hard (**B**,**D**,**F**) dough stages. The images shown illustrate true colors, DIC + red autofluorescence and red autofluorescence. Scale bar: 10 um.

**Figure 4:**
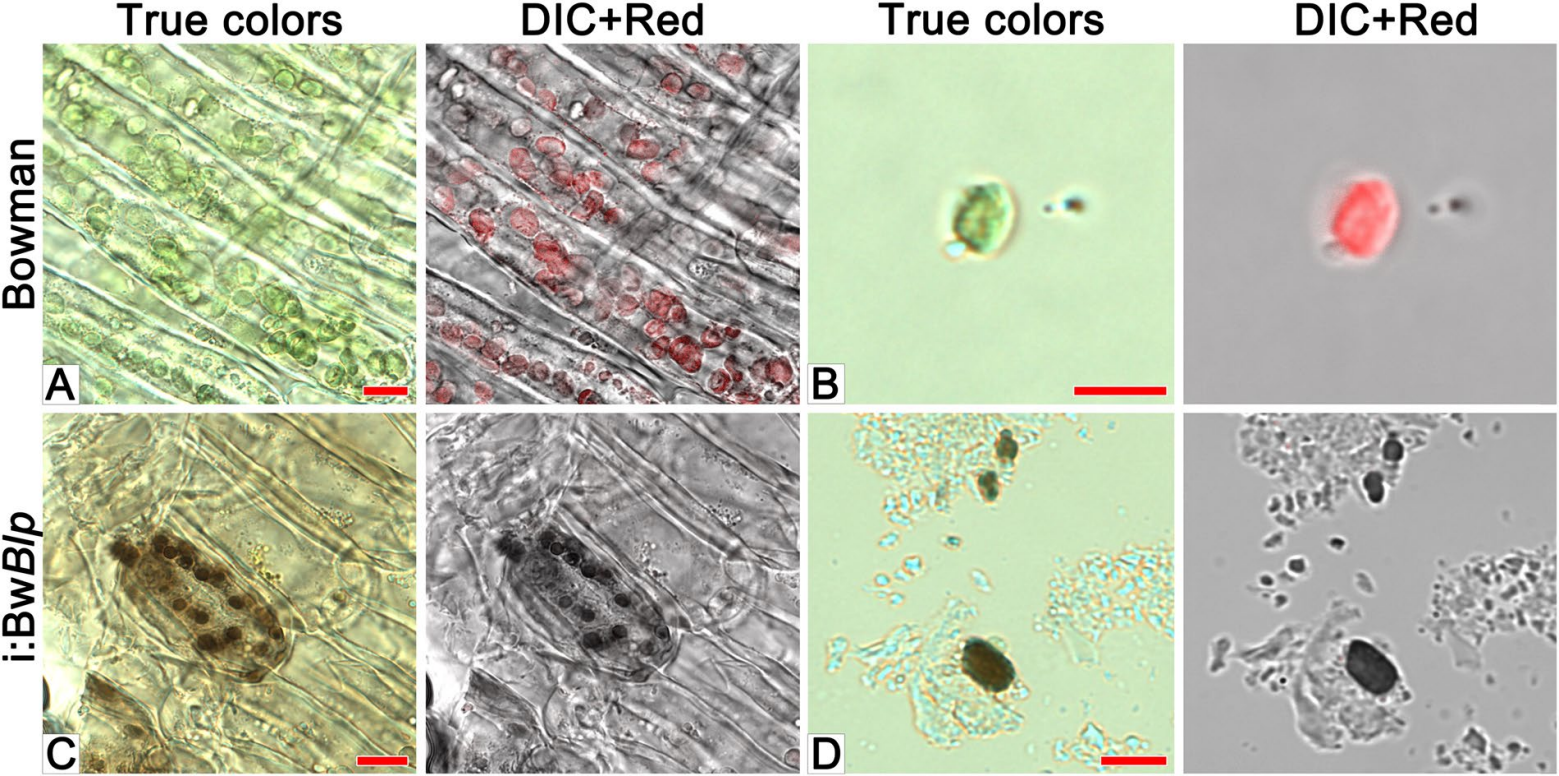
Microscopic analysis of pericarp peeled from cv. Bowman and the i:Bw*Blp* NIL, scale bar: 10 um, and isolated plastids (**B**,**D**), scale bar: 5 um. The images shown illustrate true colors, DIC + red autofluorescence.

Grains were sampled for sectioning at various developmental stages starting from the late milk stage (Fig. 2). Grains of cv. Bowman sampled at this stage exhibited a high level of red autofluorescence in the spongy parenchyma of the husk, pericarp and aleurone (layer nomenclature is presented in Supplementary Fig. S3), consistent with the presence of chloroplasts in these tissues (Fig. 2A). In the i:Bw*Blp* NIL at the early dough stage (the later one than studied in Bowman), the brown pigment was seen exclusively in the pericarp, coinciding with the location of red autofluorescence (Figs. 2D and 3A). Inspection of pericarp peeled from the grain of i:Bw*Blp* revealed that the brown color was associated with plasids which resembled the chloroplasts seen in the pericarp of cv. Bowman (Fig. 4A,C). Very little or no red autofluorescence was emitted from the pigmented areas of the i:Bw*Blp* grain. A chloroplast isolation kit was used to prepare intact plastids from the pericarp tissue of both cv. Bowman and the i:Bw*Blp* NIL (Fig. 4B,D): these were colored brown in the latter. Brown cell inclusions were observed in spongy parenchyma and bast fibres, coinciding, in a few examples, with the location of red autofluorescence (Fig. 3C,E).

In grain sampled at the soft dough stage, the red autofluorescence emitted by pericarp tissue obtained from the both NILs was less intense than in grains harvested at earlier development stages, and was barely detected in the husk (Fig. 2B,E). The i:Bw*Blp* NIL exhibited a marked irregularity in distribution of the brown pigment through the pericarp and husk tissues. In some cases, the pigments were simultaneously detected in plastids of pericarp cells and in spongy parenchyma cells, in a form of structurelles inclusions; in other grain sections, the structureless inclusions were observed in spongy parenchyma cells only (Fig. 2E); and there were grain sections with no evidence of pigmented structures as well (not shown).

By the time of the hard dough stage, the decreased red autofluorescence was recorded in pericarp of the both NILs (Fig. 2C,F). By this time, the grains set by cv. Bowman were almost colorless, with single plastids present in the pericarp; meanwhile the grains set by the i:Bw*Blp* NIL had retained a population of the brown plastids in both the pericarp and the spongy parenchyma (Figs. 2F and 3B), where the structureless inlcusions were observed too (Fig. 3D). The brown pigment was present as structureless inclusions in bast fibres cells (Fig. 3F).

### Ultrastructure of plastids

**Figure 5:**
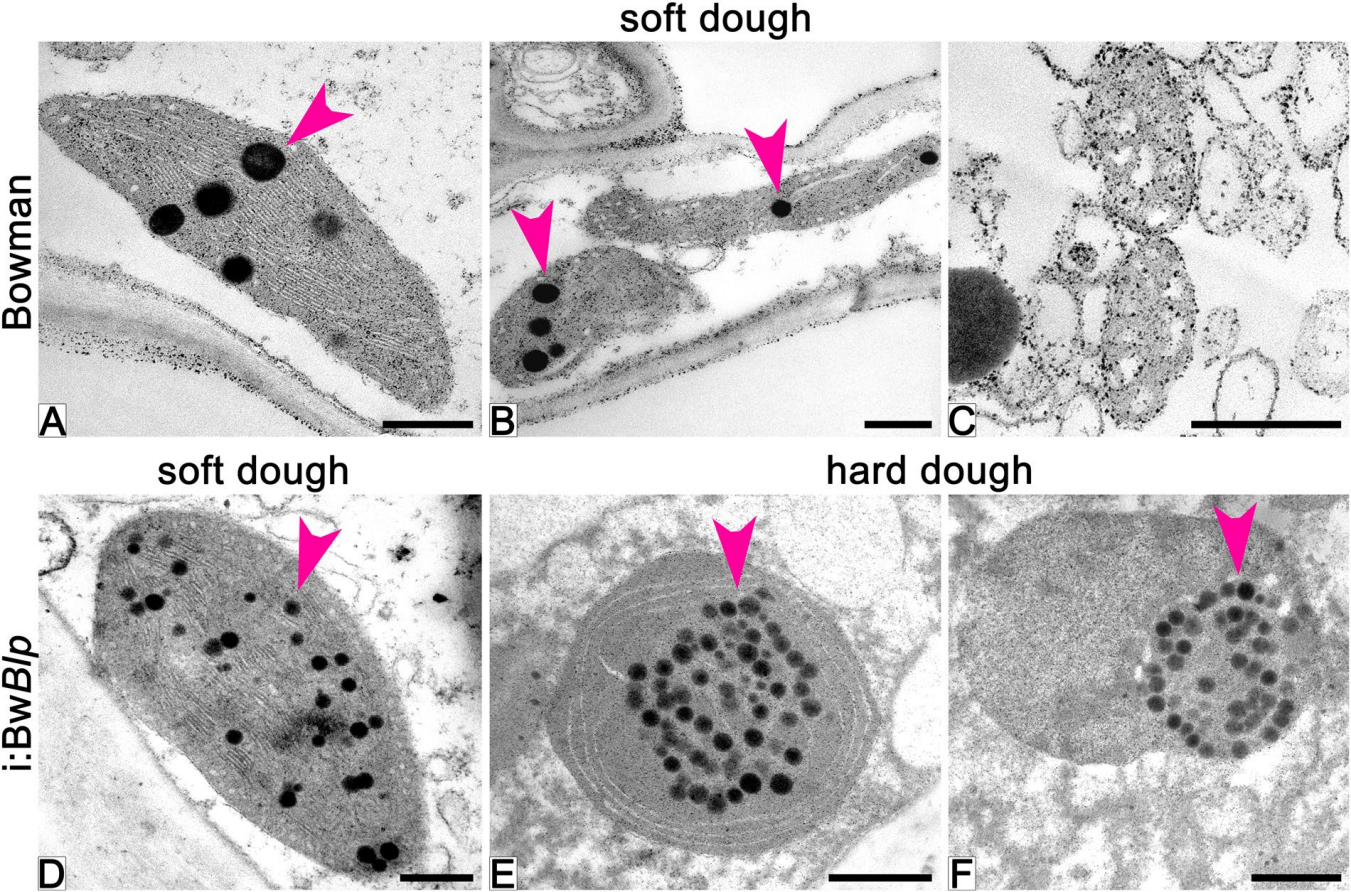
Ultrastructure of plastids in pericarp cells of cv. Bowman (**A**–**C**) and the i:Bw*Blp* NIL (**D**–**F**) at the soft (**A**–**D**) and hard (**E**,**F**) dough development stages. Arrowheads show plastoglobuli. Scale bar: 5 um.

Ultrastructure of plastids in pericarp cells of grain was studied by transmission electron microscopy (TEM) (Fig. 5). In grains of cv. Bowman sampled at the soft dough stage, part of the plastids have normal chloroplast internal structure with thylakoid stacks without any visible signs of degradation (Fig. 5A); meanwhile the majority of the plastids lose their internal structure and are assumed to be degraded (Fig. 5B,C). By the hard dough stage, cells of the pericarp almost completely lost their internal structure and organelles, which could be identify as plastids were not observed.

In grains of the i:Bw*Blp* NIL harvested at the soft dough stage, all observed plastids had normal chloroplast internal structure without visible degradation signs (Fig. 5D) which become apparent later, at the hard dough stage, when the internal membrane was organized in circle-shaped structure or was not detected at all (Fig. 5E,F). The outer membrane of the plastids persisted up to the hard dough stage, the plastid borders were obvious, and content of the plastids did not leave the organelles (Fig. 5F).

The osmiophillic particles plastoglobuli (PGs) were observed in plastids of the both NILs starting from the soft dough stage (Fig. 5). In Bowman, PGs were distributed randomly within plastids, without any tendency to aggregation, and had disappeared by the hard dough stage (Fig. 5A–C). In the i:Bw*Blp* NIL, the PGs were also distributed randomly at the soft dough stage, while they showed tendency to aggregation at the hard dough stage, after internal membrane disruption (Fig. 5E,F).

## Discussion

Melanins are synthesized by a wide range of organisms, which underlines their significance in determining evolutionary success. Despite some heterogeneity in the identity of their precursors, the end products resemble one another markedly with respect to not only their chemistry but also the pathways utilized for their synthesis. Melanins are insoluble in most organic solvents, but dissolve readily in an alkaline medium and are discolored by strong oxidizing agents; these hallmark properties have been used to devise a series of chemical tests for diagnostic use when encountering an unknown pigment^1,20,21^. These tests represented therefore the point of departure for characterizing the dark pigment purified from the husk and pericarp of the barley NIL carrying the gene *Blp* which is responsible for the formation of a black spike. The positive identification was supported by the outcome of an FT-IR spectroscopy analysis, which revealed the presence of phenolic fragments, quinone and an aromatic carbon backbone characteristic of melanin^22–25^.

When sectioned immature grains harvested from the i:Bw*Blp* NIL were compared with those harvested from cv. Bowman, it was determined that the melanin was synthesized in the pericarp within plastids exhibiting strong red autofluorescence typical for chloroplasts^26^. During grain ripening the red autofluorescence observed by light microscopy was drastically decreased in both lines. Decreasing of the red autofluorescence was coincided with the degradation of the plastid internal structure, that was clearly observed by TEM in pericarp of Bowman and the i:Bw*Blp* NIL at the soft and hard dough stages, respectively. Besides pericarp tissue, melanin was observed in the cells of the husk, where it was mostly present as structureless inclusion. The red autofluorescence in spongy parenchyma and bast fibres cells noted at the early dough stage only may indicate that the melanin in these husk tissues had initially formed in chlorophyll-containing plastids as was clearly the case in the pericarp. Then, probably after plastid degeneration during grain ripening the pigment released into cytoplasm and formed structureless melanin fusion.

Supporting the notion that melanin synthesis in the black spike barley was initiated in chloroplasts is the evidence that both the key melanogenesis enzyme PPO and the phenolic melanin substrates of PPO are present in the chloroplast^14,27,28^. PPOs (also referred to variously as tyrosinases, polyphenolases, phenolases, catechol oxidases, cresolases or catecholases) are ubiquitous in living matter. With the molluscan and arthropod oxygen carrier proteins hemocyanins PPOs belong to the type-3 copper protein family which features a binuclear active site composed of two copper atoms, each of which is coordinated by three conserved histidine residues^29^. They are thought to have evolved in response to the photosynthesis-induced shift in the atmosphere from a reducing to an oxidizing environment^30^. Although other classes of enzymes, notably laccases, peroxidases and polyketide synthases are able to oxidize phenols and thus initiate the synthesis of melanin, the PPOs are typically responsible for this process^3^. In plants, PPOs have been implicated in the browning response of damaged tissue, but their role in intact tissue is uncertain^27^. Some evidence has been presented to suggest that they participate in the synthesis of dark pigments in the rice husk^31^, but these particular pigments have not as yet been shown to be melanin.

Comparative TEM analysis of pericarp tissue of grains harvested at the early and hard dough stages showed accumulation of PGs in the both NILs which in addition to observed chloroplasts breakdown, represent characteristic feature of senescence gerontoplasts^32^. In the i:Bw*Blp* NIL, aggregated PGs persisted in plastids up to the hard dough stage, when in Bowman, neither PGs nor any plastids were detected. PGs represent lipoprotein particles surrounded by a membrane lipid monolayer with multiple functions in plastid metabolism, developmental transition and environmental adaptation^33^. PGs are characterized by their own proteome and metabolome which are different in distinct plastid types. They have striking dynamic nature and may vary substantially in form and size in response to abiotic stress or development transition^33^. The observed differences in internal structure and PGs dynamics between the NILs may be attributed to distinct metabolic processes inside these compartments leading to melanin accumulation in the case of i:Bw*Blp*, and to chloroplasts dismantling in the case of Bowman. Comparative transcriptome analysis of the husk and pericarp tissues of the same NILs demonstrated differential expression of more than a thousand genes with roles in the phenylpropanoid and fatty acid biosynthesis pathways were among the most represented and upregulated in the i:Bw*Blp* NIL^34^ supported indirectly the metabolic differences assumed between the NILs. For the new type of plastids which are assumed to accumulate melanin in PGs and to be different from well-known senescent gerontoplasts the special term “melanoplasts” is suggested.

Although the relevant underling mechanism of melanogenesis in plant remains obscure the data obtained here showed its intracellular accumulation within a membrane-delimited organelle, which is also the case in a number of other organisms. In mammalian melanocytes, synthesis takes place within a melanosome^3^, while in insects, melanin synthesis is associated with the formation of the cuticle^35^. Specialized hemocytes able to synthesize melanin in response to immune challenge have been reported in the larvae of both fruitfly^36^ and the mosquito species *Aedes aegypti*^37^ and *Armigeres subalbatus*^38^. In fungi, melanin formation has been generally associated with the cell wall, although the initial stages of its synthesis appear to be carried out in vesicles akin to mammalian melanosomes before the product is transported to the cell wall^39^. In the *Aspergillus* species *A. fumigatus* and *A. nidulas*, the enzymes involved in the initial stages of melanin synthesis are recruited by endosomes, whereas those involved in the later stages are active at the cell wall^40^. As the synthesis of melanin involves the formation of cytotoxic intermediates (quinones), there is an evolutionary advantage in compartmentalizing the process. In the barley plant, melanin appeared to be formed within senescing plastids in pericarp and husk tissues, so that any harmful effects exerted by quinones on the plastids’ photosynthetic activity would have had only a minimal impact on the plant’s overall photosynthesis. Further studies are required to clarify how melanogenesis in plants is related to photosynthesis.

## Methods

### Plant material

Grain of the pair of NILs used for the analysis were obtained from the Nordic Gene Bank (www.nordgen.org). The two lines were cv. Bowman (NGB22812) and the *Blp* carrier i:Bw*Blp* (NGB20470). Plants were raised in a greenhouse at Novosibirsk (Russia) providing a 12 h photoperiod and a temperature range of 20–25 °C.

### Extraction and characterization of spike pigment

For pigment extraction 300 g of the i:Bw*Blp* NIL’s grains were immersed for 2–4 h in cool water, after which the husk and pericarp were detached using a scalpel and immersed for 48 h at room temperature in 1.5 L 0.5 M NaOH with constant stirring. The resulting pigmented solution was filtered through cotton wadding and then through filter paper under vacuum. The pH of the filtrate was reduced to 2.0 by the addition of concentrated HCl. The black precipitate which formed as a result was rinsed in distilled water and centrifuged (3000 rpm for 20 min). The pellet was subsequently dissolved in a small volume of 0.01 M NaOH and re-precipitated with HCl, a procedure which was repeated three times. Finally, the precipitate was rinsed with distilled water, dried at 20 °C and ground to a powder in a mortar. The presence of quinoid and phenolic groups in the precipitated material was tested by its reaction with various oxidizing agents, namely H_2_O_2_^41^, KMnO_4_^42^ and FeCl_3_^43^. For the first of these reactions, an aliquot of 0.05% w/v of the powder dissolved in 0.1 M NaOH was combined with an equal volume of 10% H_2_O_2_ and left for 24 h; for the second, the 0.1 M NaOH was replaced by 0.1 M KMnO_4_, while for the third, 0.5–1.0 mg/mL FeCl_3_ was added to 0.01% w/v of the powder dissolved in 0.1 M NaOH. The solubility of the material was tested in a range of organic solvents (ethanol, isopropanol, hexane, petroleum ether, ethyl acetate, hydroxymethylformamide), in water, in concentrated (76%) H_2_SO_4_ and in 0.125 M NaOH. Fourier transform infrared (FT-IR) spectroscopy was carried out in KBr pellets using a Nicolet^TM^ 6700 FT-IR device (Thermo Fisher Scientific, Waltham, MA, USA) set to the range 4,000–400 cm^−1^. The resulting absorption peaks were interpreted as follows: 3,303 cm^−1^: -O(N)-H^25^; 2,921 cm^−1^ and 1,421 cm^−1^: -CH-^44,45^; 1,648 cm^−1^: C = C conjugated with C = O^22,46^; 1,509 cm^−1^: Car = Car (conjugated carbons in the aromatic ring)^23^; 1,240 cm^−1^ and 1,020 cm^−1^: Car-O-R(H)^24^; 1,020 cm^−1^: C-OH or C-O-C^16,45,47^.

### Cryosectioning and microscopy

Three grains were sampled at late milk (growth stage 77 based on the BBCH-scale, BBCH-77), early dough (BBCH-83), soft dough (BBCH-85) and hard dough (BBCH-87) developmental stages from each of the two NILs, snap-frozen in liquid nitrogen and stored at −70 °C until required. Prior to sectioning, the frozen grains were held at −20 °C for 30 min, mounted and embedded in Tissue-Tek O.C.T.^TM^ compound (Sakura Finetek Europe B.V., Alphen aan den Rijn, the Netherlands). Sectioning was carried out at −20 °C using an HM 505 N cryostat microtome (Microm, Walldorf, Germany). Sections of thickness 15 um were mounted on a poly-L-lysine slide (Thermo Fisher Scientific) and fixed for 15 min in 8% formaldehyde (Sigma-Aldrich, St. Louis, MO, USA) dissolved in phosphate buffered saline (pH 7.4). The slides were then rinsed twice for 15 min in distilled water, mounted in glycerol and observed under microscope. Pericarp samples were peeled from grains of both NILs at the soft dough stage, fixed in the same way as the sections, mounted on a glass slide and observed under microscope. Chloroplasts were isolated from pericarp peels using a Minute^TM^ chloroplast isolation kit (Invent Biotechnologies, Inc., Plymouth, MN, USA). The suspended chloroplasts were mounted on a glass slide and observed under microscope. Confocal laser scanning microscopy was achieved using an LSM 780 device (Zeiss, Oberkochen, Germany). Red autofluorescence was excited with a 633 nm laser. True colors were captured from sections using an AxioCam HRc camera (Zeiss).

### Ultrastructural analysis

Two grains per each NIL were sampled at the soft and hard dough stages. Tissue fragments with pericarp were cut into pieces of 2–5 mm and fixed with 2.5% ice-cold glutaraldehyde (Sigma-Aldrich, Germany) in phosphate buffer (pH 7.2) for 4 h. Then the material was washed three times for 15 min with phosphate buffer followed by postfixation with 1% osmium tetroxide (Azutite, Russia) for 4 h at a room temperature, washed with phosphate buffer three times for 15 min, and dehydrated with ethanol solutions of increasing concentrations. The samples were placed into acetone for 1 h and embedded into araldite epoxy resin (Fluka, Switzerland). Ultrathin sections with a thickness of about 80 nm were made using an Ultracut UCT (Leica, Switzerland) ultramicrotome and stained with lead citrate and uranyl acetate. The stained sections were examined using a Jeol JEM-1400 (Japan) transmission electron microscope at an accelerating voltage of 80 kV.

## Conclusion

The major finding from this investigation was that in the pigmented barley spike, melanin is synthesized in chlorophyll-containing plastids in the grain pericarp; it represents, to the best of our knowledge, the first observation of intracellular melanin production in a plant. The discovery raises questions regarding the commonality surrounding how and where melanin is synthesized, most particularly the identity of the key enzymes underlying the process and the compartmentalization of the process, which would imply an ancient and perhaps monophyletic origin of the cellular machinery involved.

## Supplementary information

Supplementary Information

## Data Availability

The extracted melanin samples are available from the corresponding author on reasonable request.
