# Supplementary material for: Melanin formation in barley grain occurs within plastids of pericarp and husk cells
Source: Sci Rep. 2020 Jan 13;10:179. doi: 10.1038/s41598-019-56982-y (PMC6957670; doi:10.1038/s41598-019-56982-y)
Supplement: Supplementary file 1 — Supplementary Information [file 41598_2019_56982_MOESM1_ESM.pdf]

Supplementary Materials

Melanin formation in barley grain occurs within plastids of pericarp and husk cells

Olesya Yu. Shoeva[1*], Sergey R. Mursalimov[1], Natalya V. Gracheva[2], Anastasiya Yu. Glagoleva[1], Andreas Börner[3], Elena K. Khlestkina[1,4]

[1] *Institute of Cytology and Genetics SB RAS, Novosibirsk, Russia*

[2] *Volgograd State Technical University, Volgograd, Russia*

[3] *Leibniz Institute of Plant Genetics and Crop Plant Research, Gatersleben, Germany*

[4] *N.I.Vavilov All-Russian Research Institute of Plant Genetic Resources, Saint-Petersburg, Russia*

*e-mail: olesya_ter@bionet.nsc.ru

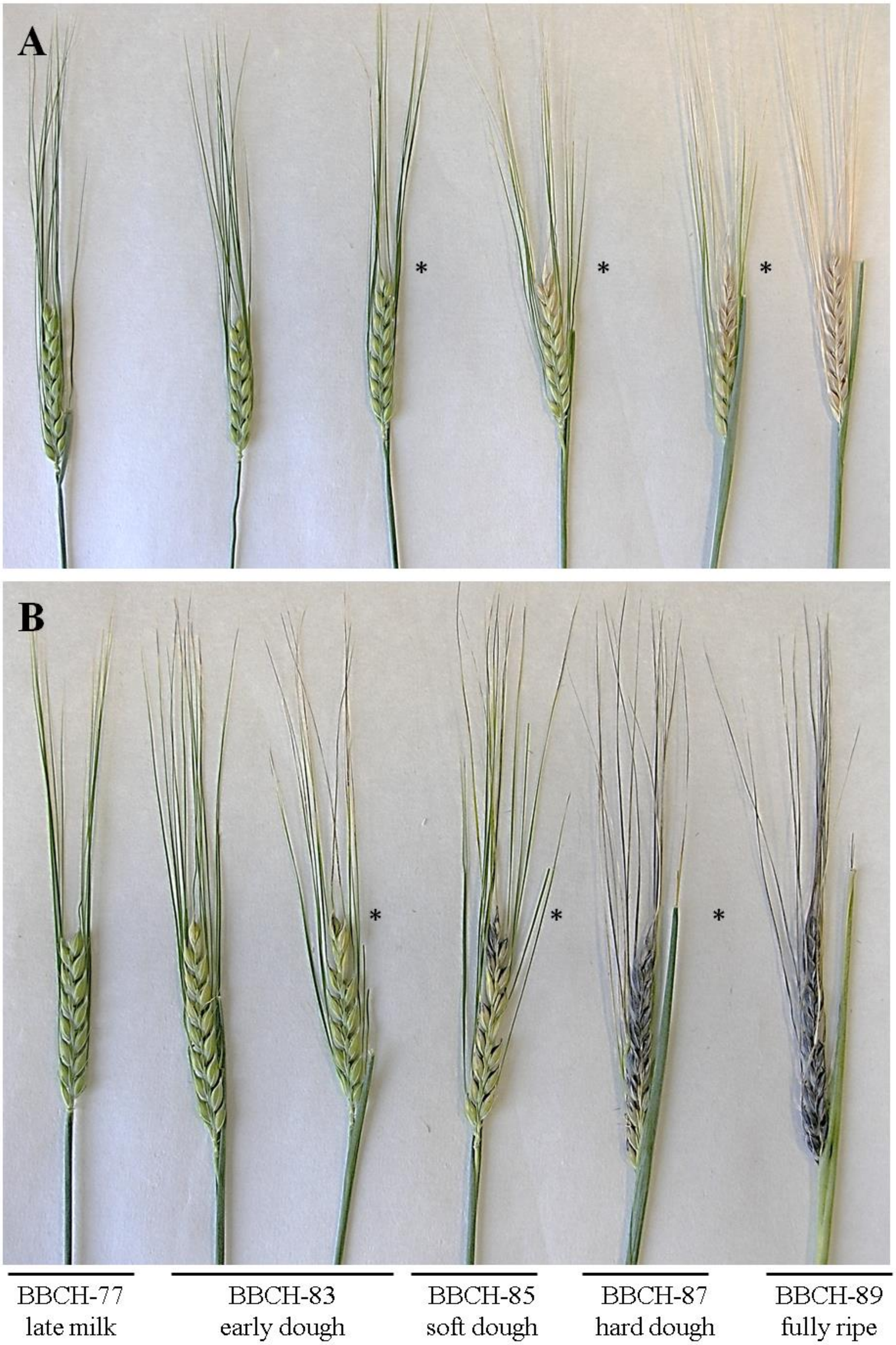

Fig. S1. Development of the spike in cv. Bowman (A) and the i:Bw*Blp* NIL (B). Asterisks mark the grain developmental stages chosen for sampling.

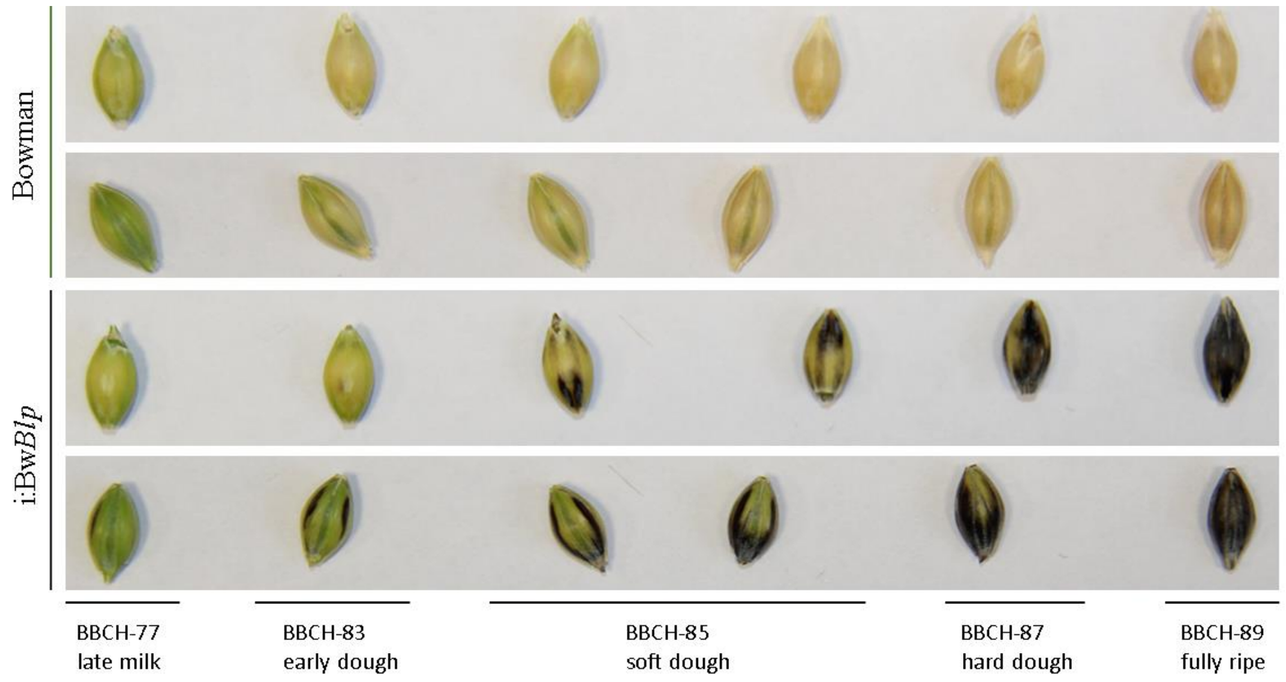

Fig. S2. Development of the grain set by cv. Bowman plants and those of the i:Bw*Blp* NIL.

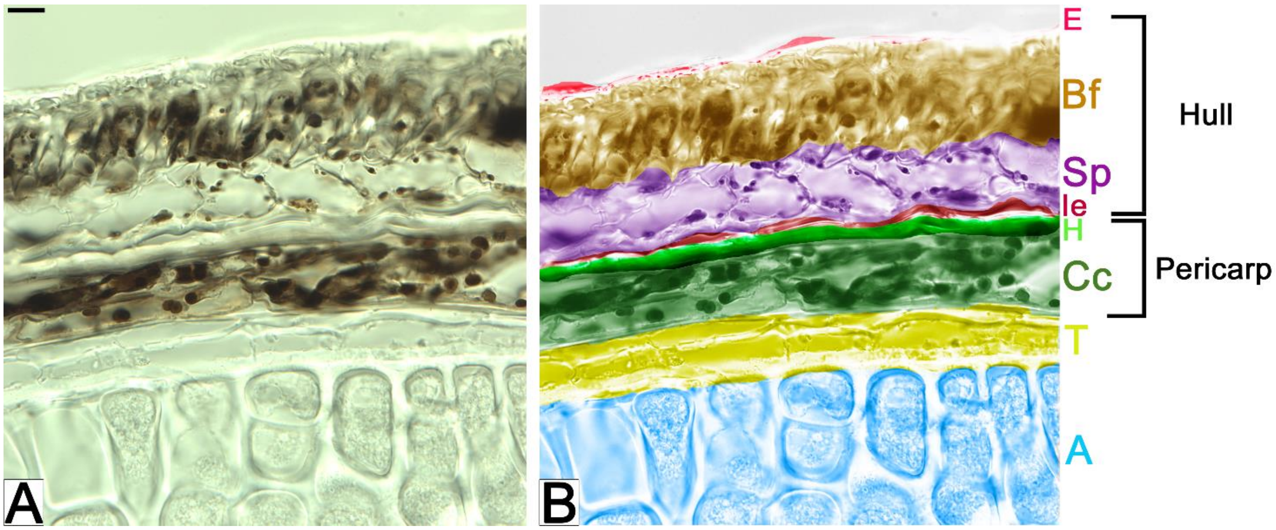

Fig. S3. Cross-section of dehusked grain of the i:Bw*Blp* NIL sampled at the hard dough stage (A) with manual dying of the grain layers using Photoshop CS2 software (Adobe Systems Incorporated) (B). A: aleurone, E: epidermis, Bf: bast fibres, Cc: cross cells, H: hypodermis, Ie: inner epidermis, Sp: spongy parenchyma, T: testa. Scale bar: 10 um.

Table S1. Solubility tests of the black pigment purified from the i:Bw*Blp* NIL. Published protocols used to solubilize anthocyanins and tannins, were ineffective in solubilizing the dark pigment from the i:Bw*Blp* husk and pericarp. Based on ability of the extracted material to dissolve in alkali solutions, discolor under the influence of strong oxidizing agents (H_2O_2, $KMnO_4$), interaction with $FeCl_3$ and resistance in common organic solvents, that have been reported as hallmarks of melanin [21], the dark pigment was referred to melanins.

Solubility	
Water and acid	
Water	Insoluble
76% H_2SO_4	Partially soluble
Alkali solutions	
0.5% NaOH	Soluble
Organic solvents	
Ethanol	Insoluble
Isopropanol	Insoluble
Hexane	Insoluble
Petroleum ether	Insoluble
Ethyl acetate	Insoluble
Hydroxymethylformamide	Swell
Oxidizing agents	
10% H_2O_2	Decolored
0.1 N $KMnO_4$	Decolored
Interaction with chemicals	
0.5-1.0 mg/ml $FeCl_3$	Precipitation al low concentration and dissolving at high concentartion

References

Sava VM, Yang S-M, Hong M-Y, Yang P-C, Huang GS (2001) Isolation and characterization of melanic pigments derived from tea and tea pholyphenols. Food Chem 73: 177–184.
